# Interferon-gamma-activated macrophages infected with Burkholderia cenocepacia process and present bacterial antigens to T-cells by class I and II major histocompatibility complex molecules

**DOI:** 10.1080/22221751.2020.1818632

**Published:** 2020-09-17

**Authors:** Roberto Rosales-Reyes, Paola Garza-Villafuerte, Daniela Vences-Vences, Daniel F. Aubert, Rubi Aca-Teutle, Vianney F. Ortiz-Navarrete, Laura C. Bonifaz, Julio Cesar Carrero-Sánchez, Alfonso Olivos-García, Miguel A. Valvano, José Ignacio Santos-Preciado

**Affiliations:** a Facultad de Medicina, Unidad de Investigación en Medicina Experimental, Universidad Nacional Autónoma de México, Mexico City, México; b Department of Microbiology and Immunology, The University of Western Ontario, London, Canada; c Departamento de Biomedicina Molecular, Centro de Investigación y de Estudios Avanzados del IPN, Mexico City, México; d Unidad de Investigación Médica en Inmunoquímica Hospital de Especialidades Centro Médico Nacional Siglo XXI, IMSS, Mexico City, Mexico; e Instituto de Investigaciones Biomédicas, Universidad Nacional Autónoma de México, Mexico City, Mexico; f The Wellcome-Wolfson Institute for Experimental Medicine, Queen's University Belfast, Belfast, UK

**Keywords:** *Burkholderia cepacia* complex, T-cell activation, antigen processing, MIIC, Rab7, Type 6 secretion, antigen presentation

## Abstract

Burkholderia cenocepacia is an emerging opportunistic pathogen for people with cystic fibrosis and chronic granulomatous disease. Intracellular survival in macrophages within a membrane-bound vacuole (BcCV) that delays acidification and maturation into lysosomes is a hallmark of B. cenocepacia infection. Intracellular B. cenocepacia induce an inflammatory response leading to macrophage cell death by pyroptosis through the secretion of a bacterial deamidase that results in the activation of the pyrin inflammasome. However, how or whether infected macrophages can process and present B. cenocepacia antigens to activate T-cells has not been explored. Engulfed bacterial protein antigens are cleaved into small peptides in the late endosomal major histocompatibility class II complex (MHC) compartment (MIIC). Here, we demonstrate that BcCVs and MIICs have overlapping features and that interferon-gamma-activated macrophages infected with B. cenocepacia can process bacterial antigens for presentation by class II MHC molecules to CD4^+^ T-cells and by class I MHC molecules to CD8^+^ T-cells. Infected macrophages also release processed bacterial peptides into the extracellular medium, stabilizing empty class I MHC molecules of bystander cells. Together, we conclude that BcCVs acquire MIIC characteristics, supporting the notion that macrophages infected with B. cenocepacia contribute to establishing an adaptive immune response against the pathogen.

## Introduction

The *Burkholderia cepacia* complex (Bcc) is a group of closely-related opportunistic bacteria associated with poor clinical outcomes in individuals with cystic fibrosis and chronic granulomatous disease [1]*.* In particular, *Burkholderia cenocepacia*, has become notorious for its ability to be transmitted across patients, often causing a fatal necrotizing pneumonia in people with cystic fibrosis [2-4].

Several studies have shown that *B. cenocepacia* can survive within macrophages in culture and in human lung mucosal tissue macrophages [5,6]. Survival occurs in a membrane-bound cytoplasmic vacuole (BcCV) [7] that delays acidification and fusion with lysosomes [8], in part due to inactivation of Rab7 [9]. In addition, intracellular *B. cenocepacia* inactivate the small GTPases Rac1 [10,11] and RhoA in a Type 6-secretion system-dependent manner [12]. Rac1 inactivation delays the assembly of the NADPH oxidase complex onto the BcCV [11,13] and affects actin remodeling, which in turn compromises normal cell migration and phagocytosis [10,11], while RhoA dysfunction, sensed by the pyrin inflammasome pathway, leads to cell death by pyroptosis [12]. Intracellular *B. cenocepacia* can also induce necrosis of human neutrophils [14,15] and alter the normal functioning of human dendritic cells by inhibiting upregulation of costimulatory molecules and inducing necrosis [16]. However, the mechanisms of cell death in both neutrophils and dendritic cells have not been investigated. *B. cenocepacia* can also mediate TNFα and IL-6 release through the stimulation of Toll-Like receptors (TLR)-4 and TLR-5 [17,18]. Macrophages are one of the first lines of defense against intracellular pathogens [19]; when activated, macrophages upregulate antimicrobial mechanisms causing bacterial lysis. Similar to dendritic cells, macrophages also act as antigen-presenting cells (APCs) by processing and displaying bacterial peptides at the cell surface, which prime T-cell immune responses [20]. Upon engulfment, bacterial antigens are processed into peptides in a late endosomal compartment (MIIC) and loaded onto newly-synthesized class II MHC molecules [21]. The class II MHC/peptide complexes assembled in the MIIC are transported to the plasma membrane, where they can be recognized by specific CD4^+^ T-cells to initiate an adaptive immune response [22]. Further, antigens in the cytosol of an APC are processed into peptides by the proteasome [21]; these are subsequently translocated into the endoplasmic reticulum by the transporter antigen processing protein 1 and 2 (TAP1/TAP2). In the endoplasmic reticulum, the translocated peptides are loaded onto newly-synthesized class I MHC molecules. The class I MHC/peptide complex is transported to the plasma membrane and recognized by specific CD8^+^ T-cells [21]. APCs could also use an alternative pathway to present exogenous antigens that reside in vacuoles, which involves the release of processed peptides from vacuoles into the extracellular medium where they can interact with empty class I MHC molecules of the same cell or bystander cells [23].

Although several mechanisms triggering innate immune responses upon *B. cenocepacia* infection have been described, whether infected macrophages prime the adaptive immune system has remained unexplored. The intracellular infection of macrophages with *B. cenocepacia* alters the normal functions of these cells [10,11]. Because the bacteria reside within a modified vacuole that delays the fusion with the lysosomes and the MIIC is an intracellular compartment that does not necessarily interact with lysosomes, we speculated that the BcCV shares features with the MIIC and wanted to investigate whether and how *B. cenocepacia*-infected macrophages can present antigens to T-cells. Here, we show that BcCVs and the MIIC acquire similar markers and *B. cenocepacia*-infected macrophages can present bacterial antigens by class I and class II MHC molecules to primed T-cells, suggesting that the infected macrophages, despite being functionally impaired in some respects, can still contribute to establish an adaptive immune response against *Burkholderia*.

## Materials and methods

### Ethics statement

Mice C57BL/6 and C3H/HeJ were obtained from Unidad de Investigación en Medicina Experimental and the Instituto de Investigaciones Biomédicas, Universidad Nacional Autónoma de México, respectively. Animals were maintained under Institutional Animal Care and Use Committee guidelines described in the NOM-062-ZOO-1999. This protocol was approved by the Research and Ethics committee of the School of Medicine, National Autonomous University of Mexico # FMED/CI/JMO/008/2014.

### Bacterial strains and growth conditions

Bacteria and plasmids used in this study are described in Supplementary Table 1. In this study, we employed the *B. cenocepacia* strain MH1* *K (herein *B. cenocepacia*), which carries a deletion of an efflux pump that makes it sensitive to aminoglycoside antibiotics, enabling us to carry out accurate determinations of intracellular bacterial survival by differential killing of extracellular bacteria with gentamicin [24] (see below). *Escherichia coli* and *B. cenocepacia* were grown in Luria–Bertani broth (LB; Sigma-Aldrich) overnight at 37*°*C with shaking (180* *rpm). Plasmids in *Escherichia coli* were mobilized into *B. cenocepacia* by triparental mating [25]. *B. cenocepacia*(pDSRedT3) was grown in LB supplemented with 30* *μg/ml chloramphenicol, while *B. cenocepacia*(p*zmpA*-HEL_48-61_) or *B. cenocepacia*(p*zmpA*-OVA_257-264_) were grown in LB supplemented with 50* *μg/ml trimethoprim. Once introduced in *B. cenocepacia* plasmid-encoded fusion proteins were expressed by induction with 0.2% (w/v) rhamnose (Sigma-Aldrich) during 4* *h at 37*°*C with shaking.

### Construction of recombinant plasmids

To determine antigen presentation to CD4^+^ and CD8^+^ T-cells, we constructed two plasmids (Supplementary Table 1). Briefly, we used pJLP1E (Crl-OVA) that encodes the *crl* gene fused to a DNA fragment encoding an ovalbumin peptide (OVA_254-267_) and pJLP2H (Crl-HEL) encoding the *crl* gene fused to a DNA fragment encoding a hen egg lysozyme peptide of the (HEL_48-61_). Both plasmids were kindly donated by Dr. Mary Jo Wick (University of Gothenburg, Sweden) and confirmed by DNA sequencing. The HEL_48-61_ coding sequence was amplified with the HEL-Xba (Fw) 5'-TTTTTCTAGACGTAACACCGATGGGAGTAC-3’ and HEL-HindIII (Rv) 5'-TTTTAAGCTTCCTGCCATCGTTAACCCATCA-3’ primer pairs, and the OVA_254-267_ coding sequence was amplified with the OVA-Xba (Fw) 5'-TTTTTCTAGATCTATAATCAACTTTGAAAAAC-3’ and OVA-HindIII (Rv) 5'-TTTTAAGCTTTTTCACTCACGCCGTTATCAC-3’ primers. PCR products were cloned into pSCRha behind the rhamnose-inducible promoter [26], resulting in pSCR-HEL_48-61_ and pSCR-OVA_257-264_. Next, we amplified the *Burkholderia zmpA* gene using primers 5647 and 5648 [26] to construct plasmids encoding ZmpA protein fusions to HEL_48-61_ and OVA_257-264_. A haemagglutinin tag was also incorporated into these fusions to assess protein expression by western blot. The resulting plasmids, pSCR-ha-ZmpAHEL_48-61_ (pDA195) and pSCRha-ZmpAOVA_254-267_ (pDA196) were mobilized into *B. cenocepacia* as described above.

### Antibodies

The rat monoclonal antibody anti-LAMP1 (clone ID4B) was obtained from Beckton Dickinson, the rabbit anti-Rab7 (H-50) was from Santa Cruz Biotechnology, the mouse anti-H-2K^b^ clone Y3 was a gift from Dr. Gunter J. Hammerling, German Cancer Research Center, Heidelberg, Germany. Armenian hamster anti-CD80 (clone 16-10A1) conjugated to PE, rat anti-CD86 (clone GL1) conjugated to FITC, rat anti-MHC-II (I-A/I-E, clone M5/114), and mouse anti-H-2Kb/pOVA_257-264_ (SIINFEKL) conjugated to PE were from Thermo Scientific. AF-488-conjugated chicken anti-mouse and AF-488-conjugated goat anti-rat (Invitrogen) were used as secondary antibodies

### Bone marrow-derived macrophages and infection conditions

Bone marrow-derived macrophages (BMDM) were obtained from femurs of C3H/HeJ or C57Bl/6 mice, as previously described [27]. Briefly, 3 x10^5^ macrophages from C57Bl/6 were seeded on 12-well plates; cells were activated with 300 U/ml of interferon gamma (IFNγ) of 10* *ng/ml of lipopolysaccharide (LPS) (both from Sigma-Aldrich) for 24* *h, as previously reported [28,29]. *B. cenocepacia* was grown overnight in LB at 37°C with shaking. One millilitre of culture was washed twice with PBS and resuspended in 1* *ml of RPMI 1640. On the day of infection, the medium was replaced with fresh RMPI without antibiotics. *B. cenocepacia* were added to macrophages and plates were centrifuged for 1* *min at 1200* *rpm, followed by incubation at 37*°*C under 5% CO_2_ and 5% humidity for the desired time. At 1* *h postinfection, cells were washed 3 times with PBS to remove extracellular bacteria and placed in fresh medium supplemented with 50* *μg/ml gentamicin (Sigma-Aldrich). To determine colony-forming units (CFUs), cells were lysed with PBS-Triton X-100 2% (Sigma-Aldrich). Aliquots of 10-fold serial dilutions of cell lysates were plated on LB-agar to quantify the number of CFUs. The percent intracellular survival was calculated as % survival = (CFUs[24* *h]×100)/CFUs[1* *h].

### Immunofluorescence and flow cytometry assays

For immunofluorescence, BMDM from C57Bl/6 mice were seeded onto square coverslips in six-well tissue culture plates. *B. cenocepacia*(pDSRedT3) was added at a multiplicity of infection (MOI) of 50 and the plates were centrifuged for 1* *min at 1200* *rpm. Infected BMDM were washed 3 times with PBS and incubated at 37°C under 5% CO_2_ with fresh RPMI 1640 supplemented with 50* *μg/ml gentamicin for the desired times. Infected macrophages were washed three times with PBS and fixed for 30* *min with 2% paraformaldehyde at room temperature; then, cells were washed twice with PBS and incubated 20* *min with 100* *mM glycine at room temperature. After fixation, macrophages were permeabilized with cold methanol for 1* *min and incubated for 1* *h in blocking buffer (PBS with 2% goat serum and 3% of BSA). Samples were then incubated for an additional hour at 37°C with the primary antibody, washed three times, and incubated with the secondary AF488-conjugated antibody for 45* *min at 4*°*C in blocking buffer. Coverslips were mounted on glass slides using fluorescent mounting medium (Dako, Cytomation). For phagocytosis assays, macrophages were incubated with 1μg/ml of AF488-conjugated zymosan particles (Molecular Probes) for 15* *min, washed three times, and then fixed and mounted on glass slides with fluorescent mounting medium. Acidification of BcCVs in *B. cenocepacia*-RFP-infected macrophages was examined with 1μM LysoTracker Green (Molecular Probes) at 30* *min post-infection. Fluorescence was assessed using a Nikon NE300 inverted microscope; images were analysed by Metamorph® (Molecular Dynamics, Downington, PA, USA). The colocalization between *B. cenocepacia*-RFP and cellular markers was evaluated by quantifying pixels in the red and green channels of every acquired image and determining the Pearson’s correlation coefficient (PCC) [30].

For flow cytometry, macrophages from C57Bl/6 mice incubated with zymosan particles or infected with *B. cenocepacia* were harvested in cold PBS with 0.1* *mg/ml EDTA. Infected cells were incubated in blocking buffer in a 96-well plate for 45* *min on ice. The cells were centrifuged and incubated in blocking buffer with anti-CD80, anti-CD86, anti-MHC-I (Y3), anti-MHC-I (H-2K^b^/pOVA) and anti-MHC-II, respectively. Stained macrophages were analysed by forward and side scatter; a region in which macrophages are located (excluding cell debris) was selected and cells were analysed by detecting specific molecules in green and red channels. The autofluorescence was compensated in relation to unstained cells. Fluorescence readings were acquired in an LSRF-Fortessa BD flow cytometer and samples analysed with the FlowJo software.

### Antigen presentation assays

Interferon-gamma (IFNγ)-activated macrophages from C3H/HeJ (haplotype I-A^k^) mice (3 x10^5^ cells) were seeded onto 12-well tissue culture plates one day before the infection. As a positive control, we incubated the cells with 250* *μg/ml of HEL protein (Sigma-Aldrich). At the same time, cells were infected with live or heat-killed *B. cenocepacia*-p*zmpA*-HEL_48-61_ at 37*°*C with a MOI of 50 for 1* *h and extracellular bacteria removed by washing twice with PBS. Infected cells and uninfected cells incubated with the HEL protein were co-cultured with C-10 hybridoma (1:2 ratio) [31] for 24* *h. Cell supernatants were collected and analysed for interleukin-2 (IL-2) by ELISA. We quantified the IL-2 released by C-10* *T hybridoma after the recognition of the MHC-II/peptide (I-A^k^/HEL_48-61_) complex to evaluate APC mediated T-cell activation [31,32]. For detection of antigen presentation by MHC-I, we infected 3 x10^5^ IFNγ-activated macrophages from C57Bl/6 (haplotype H-2K^b^) as described above. The infected cells were co-cultured with RF33.70 hybridoma (1:2 ratio) [33] for 24* *h; then, supernatants were collected and analysed by IL-2 release by ELISA. CD8^+^ T-cell activation was assessed by quantifying IL-2 release by RF33.70 after the recognition of MHC-I (H-2K^b^/pOVA_257-264_) complex.

### Co-culture assays

Three x 10^5^ IFNγ-activated macrophages from C3H/HeJ mice were seeded onto 12-well tissue culture plates one day before the infection. The cells were incubated with live or heat-killed *B. cenocepacia* at 37*°*C at a MOI of 50 for 1* *h and then washed twice with PBS. Infected macrophages were co-cultured with RMA-S cells (TAP 2^-/-^ at a 1:1 ratio) [23] in RMPI supplemented with 100* *μg/ml gentamicin for 24* *h. RMA-S cells were subsequently harvested and stained with anti-K^b^ monoclonal antibody (Y3 antibody). Fluorescence readings were acquired in an LSRF-Fortessa BD flow cytometer and analysed with the FlowJo software.

### Phagosome purification and biochemical analyses

For the purification of phagosomes from macrophages infected with *B. cenocepacia* we employed the peritoneal macrophage-like cell IC21 obtained from C57Bl/6 mice (TIB-186 from ATCC, haplotype H-2K^b^) [34]. Phagosomes were purified as previously described [11] with minor modifications. Briefly, 1 *× *10^8^ macrophages were infected with *B. cenocepacia* at a MOI of 50 for 1* *h and broken in a Dura-Grind stainless-steel homogenizer (Wheaton Scientific), as described [11]. Post-nuclear supernatants (PNS) obtained after centrifugation at 700 RPM for 2* *min were adjusted to 39% (w/v) sucrose (2* *ml of PNS plus 2.4* *ml of 65% sucrose, from Sigma-Aldrich). A step-sucrose gradient was prepared (Beckman) and centrifuged for 1* *h (28000* *rpm at 4°C) using a SW41Ti rotor (Beckman-Coulter) [11]. Ten-μl of each fraction was mixed with 90* *μl of PBS plus 1% Triton X-100, the lysates were plated on LB-agar. Colony-forming units were counted after 24* *h incubation at 37°C. Lysosomal activity was estimated by measuring β-galactosidase. For this, 25* *μl of each fraction was mixed with 125* *μl of 10* *μM of *p*-nitrophenyl-β-D-galactopyranoside/0.7% Triton X-100/150* *mM citrate buffer (pH 3.5) and incubated for 2* *h at 37°C. The reaction was stopped by addition of 150* *μl of 0.5 M of sodium carbonate, and the absorbance at 405* *nm was measured in a spectrophotometer. The protein concentration in each fraction was determined by the Bradford assay (Bio-Rad). To detect LAMP1, 15* *μl of each fraction was loaded on a 14% SDS-PAGE gel and transferred to nitrocellulose membranes. The membranes were incubated overnight at 4°C in 10% of blocking solution (Bio-Rad) made in PBS-Tween 20 (0.1%) (PBS-T) and were incubated with 1:100 of anti-LAMP1 overnight at 4°C in 5% of blocking solution in PBS-T. After washing with PBS-T, the blots were incubated with goat anti-Rat conjugated to IRDye 800CW (LICOR, Odyssey) for 1* *h at room temperature in 5% blocking solution in PBS-T. Unbound antibodies were removed by several washes with PBS-T, and the membranes were analysed by infrared imaging using an Odyssey imager (LICOR Odyssey).

### Cytotoxicity assay

Supernatants of uninfected or infected macrophages from C57Bl/6 mice with *B. cenocepacia* were assayed for released lactate dehydrogenase (LDH; Promega, Madison WI) activity. The percent LDH release was determined as percent of release = [experimental LDH release – spontaneous LDH release]/[maximal LDH release – spontaneous LDH release]×100% [27].

### Statistical analysis

Data are shown as mean ± the standard deviation (SD). Statistical analyses were conducted with GraphPad Prism 7.0. One-way analysis of variance (ANOVA) and Tukey test for multiple comparisons were performed. Data analyses were performed with a minimum of three biological repeats, each with at least two technical repeats. Asterisks indicate statistical significance and the *p* values are indicated as *, *p*<0.05; **, *p*<0.01 and ***, *p*<0.001; ns, non-significant.

The statistical analysis of colocalization between the BcCV and cellular markers was conducted by quantification of the Pearson’s correlation coefficient (PCC) with the following formula:

$$\mathrm{PCC}=\frac{\Sigma_{i}(R_{i}-\bar{R})\times (G_{i}-\bar{G})}{\sqrt{\Sigma_{i}{\left(R_{i}-\bar{R}\right)}^{2}\times {\left(G_{i}-\bar{G}\right)}^{2}}}$$

in where, *R_i_* and *G_i_* indicates the intensity values in pixel of the signal in red and green channels respectively. $\bar{R}$ and $\bar{G}$ indicates the mean intensities of the red and green channels respectively across the entire image. A PCC of 1.0 (100% of colocalization) indicates a strong correlation and a PCC of 0.0 indicates non-correlation (0.0% of colocalization) [30].

## Results

### IFNγ-treated macrophages control *B. cenocepacia* infection

Efficient antigen processing and presentation to T-cells requires activation of APCs. Usually, APC activation with IFN-γ upregulates the expression of class I and class II MHC molecules, cathepsins, TAP1 and TAP2 chaperones, proteasomal proteins, and the CD80 (B7.1) and CD86 (B7.2) co-stimulatory molecules [35–37]. Intracellular *B. cenocepacia* causes defects in macrophage function affecting actin cytoskeleton remodeling, phagocytosis, Rho-GTPases functionality, delayed assembly of the NADPH dehydrogenase, and pyroptosis [8–13,38] However, the effect of intracellular *B. cenocepacia* on IFNγ-treated macrophages in relation to antigen processing and presentation has not been investigated. Resting and IFNγ-treated macrophages were infected with *B. cenocepacia* and the expression of class I and II MHC molecules, CD80, and CD86 was quantified at 24* *h post-infection. LPS-treated macrophages infected with *B. cenocepacia* were used as a positive control for macrophage activation.

**Figure 1.**
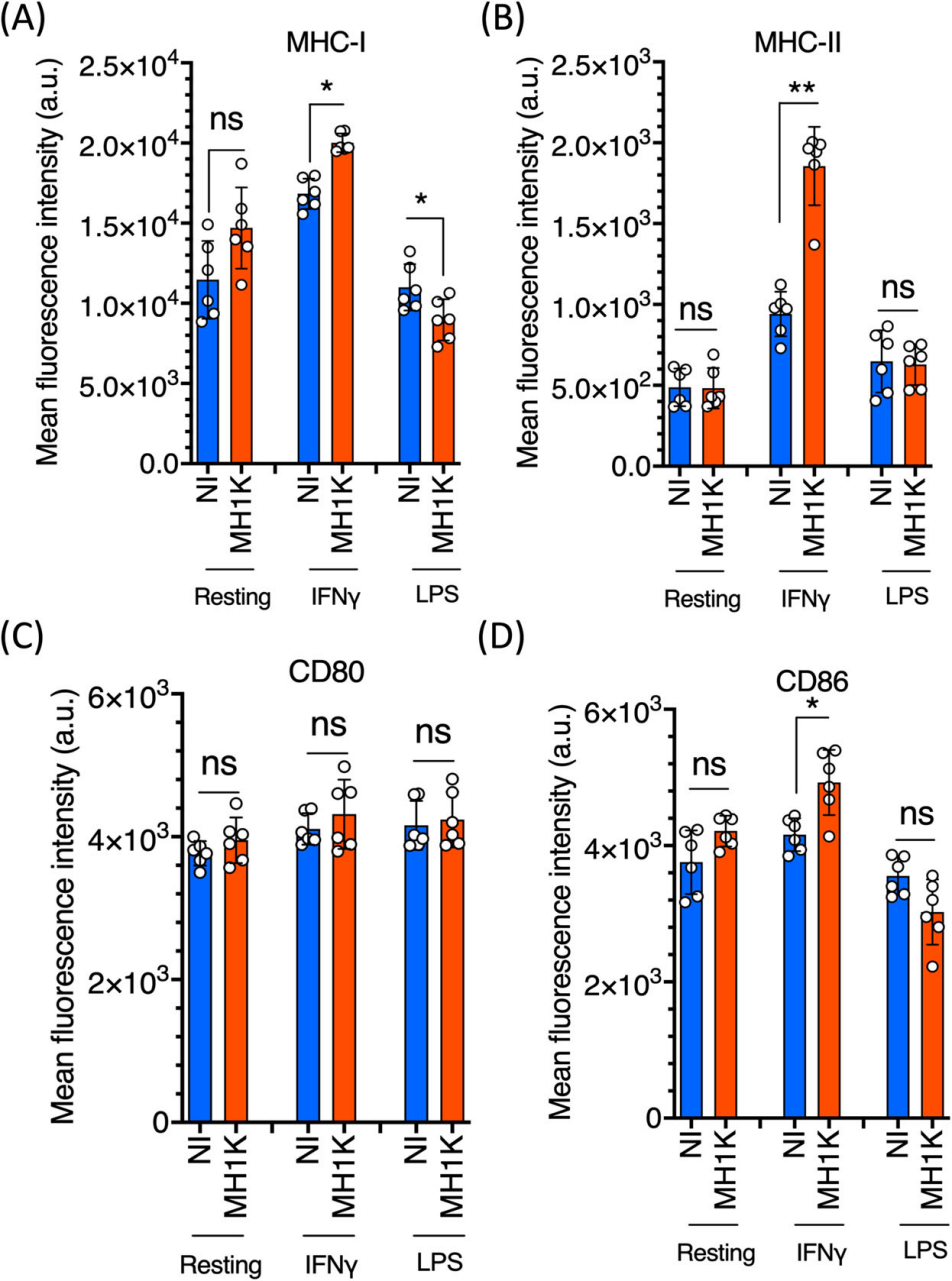
Macrophages infected with B. cenocepacia increased the expression of MHC and CD86 molecules. Resting macrophages or macrophages pre-treated with 300 U/ml of IFN*γ* or with 10 ng/ml of LPS from C57Bl/6 mice were infected with B. cenocepacia (MH1 K) at a MOI of 50 for 1 h. Cells were washed, incubated for 24 h and processed for flow cytometry analysis. Cell populations were analyzed by the fluorescence intensity of class I MHC (A), class II MHC (B), CD80 (C) and CD86 (D) molecules. Blue bars indicate expression in non-infected macrophages and the orange bars the level of expression in infected (MH1 K) macrophages. The results were obtained from 3 independent experiments, each one in duplicate (*n* = 6), and plotted as the mean fluorescence intensity ± SD and analyzed by the paired t-test. NI, non-infected; a.u., arbitrary units; *, *p*<0.05; ns, non-significant.

**Figure 2.**
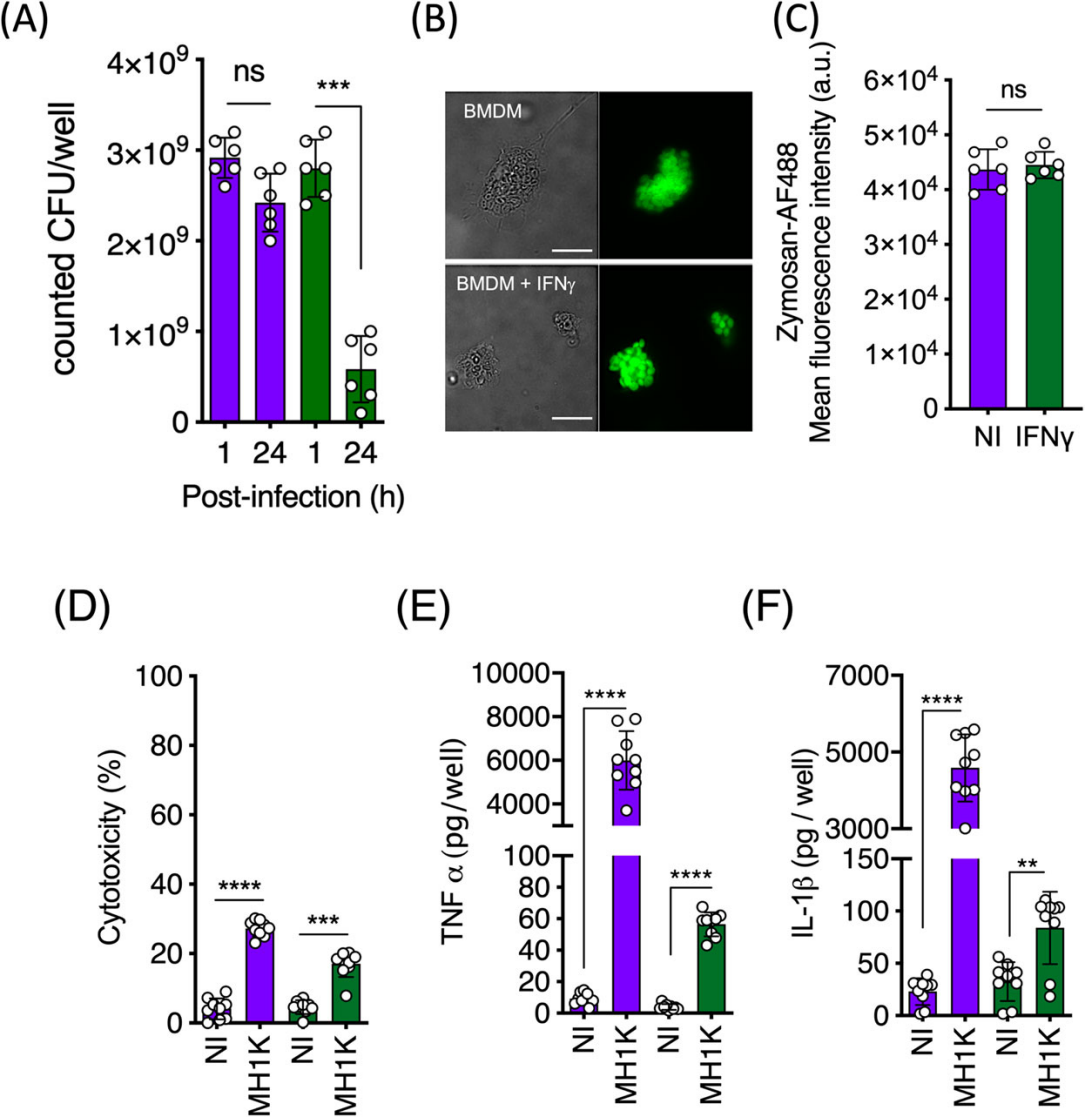
IFN*γ*-treated macrophages control intracellular *B. cenocepacia.* Three x 10^5^ macrophages from C57Bl/6 mice were pre-treated with 300 U/ml of IFN*γ* and infected with *B. cenocepacia* at a MOI of 50. (A) Non-treated macrophages (purple bars) or pre-treated macrophages with IFN*γ* (green bars) were infected with *B. cenocepacia* MH1 K by 1 h, the cells were washed and lysed at 1 or 24 h post-infection to quantify the number of bacterial CFUs per well. (B) Non treated (BMDM) or pre-treated macrophages with IFN*γ* (BMDM + IFN*γ*) were incubated with 1μg/ml of zymosan particles conjugated to AF488 during 15 min. Cells were analyzed by immunofluorescence microscopy. Bars indicates 10 μm. One representative image of 15 is presented. (C) Cells were analyzed by flow cytometry. Non-treated macrophages (purple bar) or pre-treated macrophages with IFN*γ* (green bar), one representative experiment of three replicates (*n*=6) is presented. 20000 events were analyzed by each experiment. (D) Culture supernatants were used to quantify macrophage cell death (cytotoxicity) by the assaying released lactate dehydrogenase activity at 24 h post-infection. Released TNFα (E) and IL-1β (F) at 24 h post-infection were detected by ELISA on macrophages non-infected (NI) or infected with *B. cenocepacia* (MH1 K). Green bars (D, E and F) indicate IFN*γ*-treated macrophages and the purple bars indicate resting macrophages. The results were obtained from 3 independent experiments, each one in triplicate (*n* = 9), plotted as mean ± SD and analyzed by the paired test. **p<0.01; ****p<0.0001; ns, non-significant.

In LPS-treated macrophages from C57Bl/6 mice, the intracellular infection was associated with a modest but significant decrease in the expression of class I MHC molecules at 24* *h post-infection (Figure 1(A)) but it did not significantly affect the expression of class II MHC, CD80 and CD86 costimulatory molecules (Figure 1(B–D)). In contrast, IFNγ-treated macrophages showed increased expression of class I and class II MHC molecules and CD86, while CD80 expression did not change (Figure 1). These differences were not related to variations in bacterial uptake since in resting and IFNγ-treated macrophages the uptake of *B. cenocepacia* was similar (Figure 2(A)). Further, resting or IFNγ-treated macrophages engulfed Zymosan particles in similar proportions (Figure 2(B,C)). Together, these results indicate that IFNγ-treated macrophages infected with *B. cenocepacia* are primed to process bacterial antigens.

We also evaluated whether IFNγ-activated macrophages clear the *B. cenocepacia* intracellular infection by determining bacterial colony forming units (CFUs) at 1 and 24* *h post-infection. Given that *B. cenocepacia* replicates poorly in macrophages and its generation time is approximately 1* *h in culture medium [24], the CFUs at 1* *h post-infection denote bacterial uptake, while the values at 24* *h post-infection denote bacterial survival. In contrast to untreated macrophages, which showed a 15 ± 9% reduction in the bacterial load over 24* *h (Figure 2(A)), IFNγ-activated infected macrophages had a 75 ± 6% reduction (Figure 2(A)). IFNγ-activated macrophages remained viable at 24* *h post-infection (Figure 2(D)) and retained their ability to release pro-inflammatory cytokines to the culture supernatant (Figure 2(E,F)).

### Macrophages infected with B. cenocepacia process and present bacterial antigens to CD4^+^ T cells

**Figure 3.**
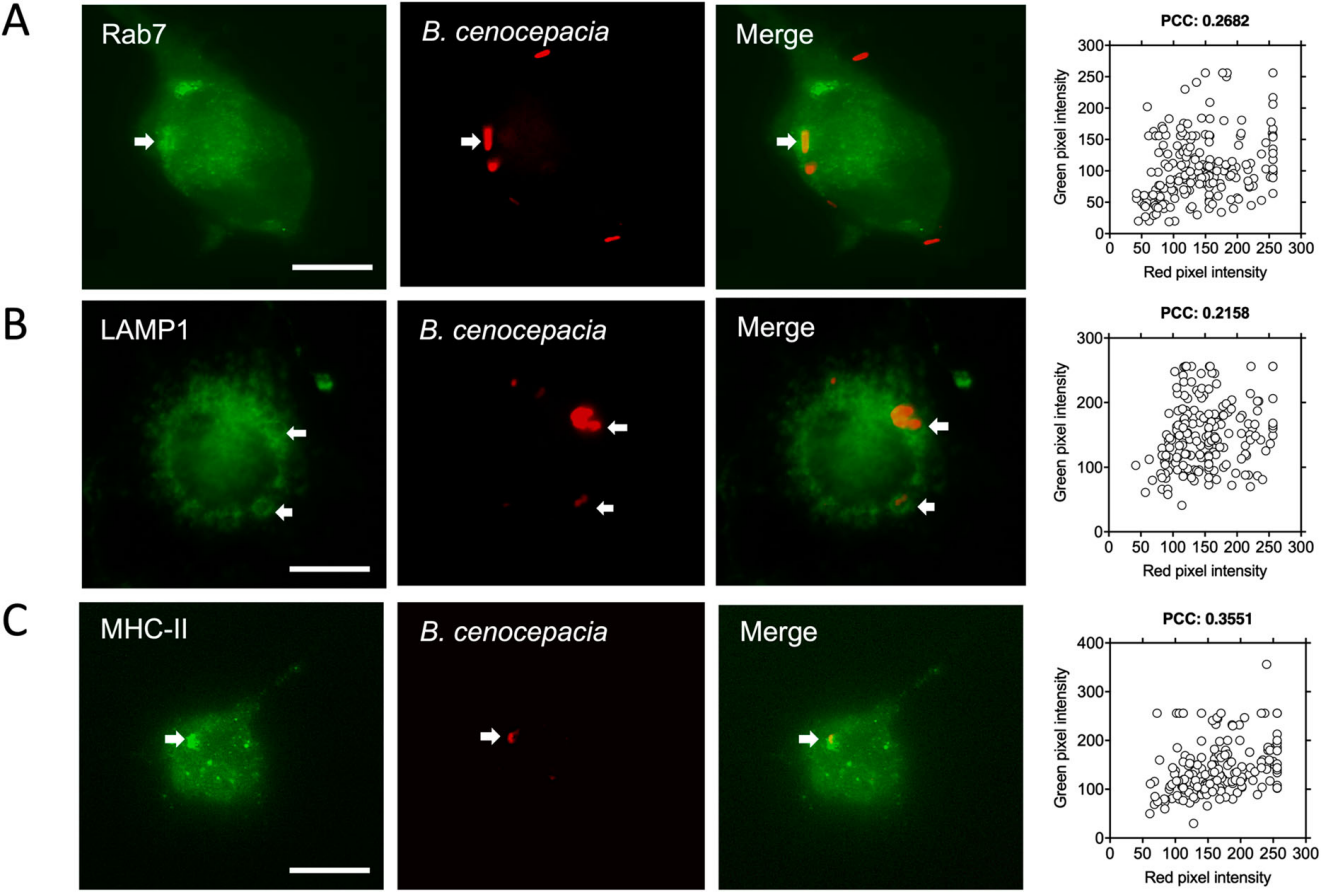
Intracellular *B. cenocepacia* is localized in the MHC class II compartment. IFNγ-treated (300 U/ml) macrophages from C57Bl/6 mice were incubated with *B. cenocepacia* (pDSRedT3) at a MOI of 50 during 1* *h. At 3* *h post-infection, infected macrophages were stained with anti-Rab7 (A), anti-LAMP1 (B) or anti-class II MHC (C). Images of infected macrophages were analyzed by immunofluorescence. Bars indicates 10* *μm. For Rab7 we analyzed 202 BcCV in 141 cells; for LAMP1, 192 BcCV in 128 cells and for MHC class II, 181 BcCV in 132 cells. The co-occurrence of green and red pixels was determined by Pearson’s correlation coefficient (PCC).

**Figure 4.**
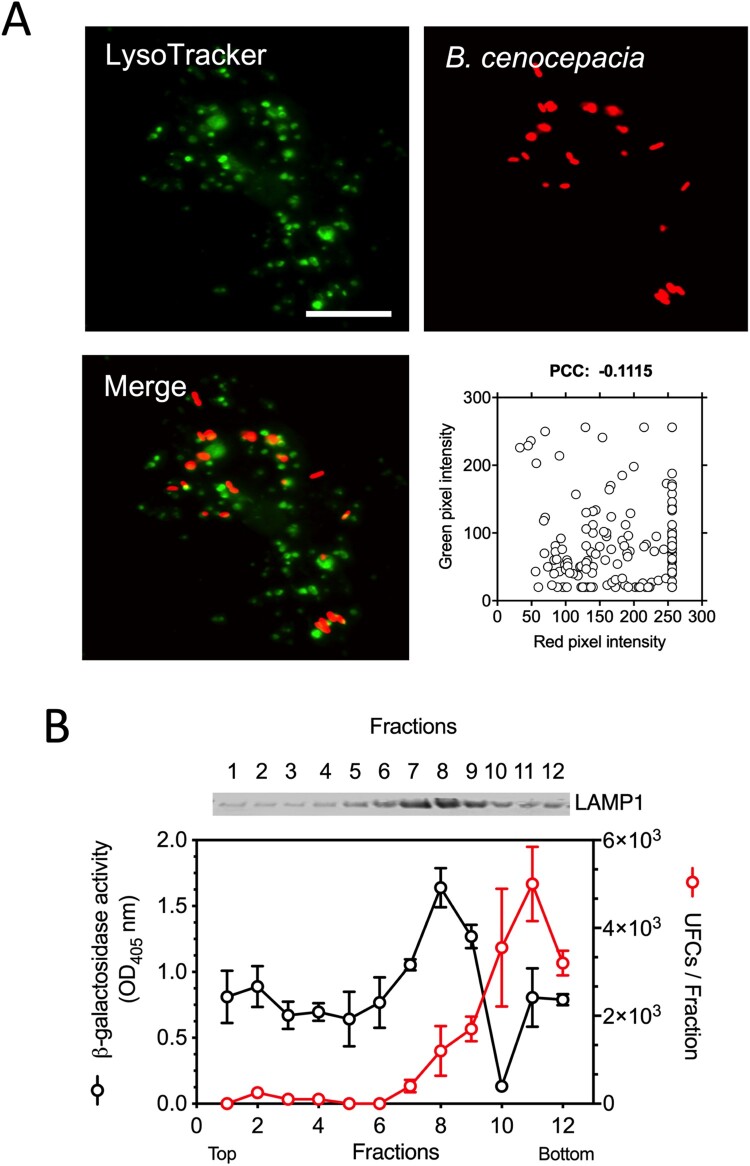
The BcCV is a non-acidic compartment. IFNγ-treated (300 U/ml) macrophages from C57Bl/6 mice were infected with *B. cenocepacia* (pDSRedT3) at a MOI of 50 during 1* *h. At 3* *h post-infection, infected macrophages were incubated with LysoTracker Green 1* *μM (A). Images of infected macrophages were analyzed by immunofluorescence. Bars indicates 10* *μm. The co-occurrence of green and red pixels was determined by Pearson’s correlation coefficient (PCC). (B) Sucrose gradient purification of vacuoles from IC-21-like peritoneal macrophages from C57Bl/6 mice infected with *B. cenocepacia*. The CFUs (Black dots) and β-galactosidase activity (Red dots) were quantified from each gradient fraction. The expression of LAMP1 was determined by western blot. The average of two biological repeats are presented. Bottom correspond to the gradient fractions with high density and the Top to the gradient fractions with low density.

Previous work demonstrated that intracellular *B. cenocepacia* reside in the BcCVs, which are late endosomal compartments that fuse partially with lysosomes [8]. Interestingly, the intracellular compartment in which antigenic peptides are generated from exogenous antigen processing (MIIC) has low lysosomal enzyme activity and is typically decorated with MHC class II molecules and Rab7 [39,40]. Therefore, we investigated if the BcCVs contain class II MHC molecules, LAMP1 and Rab7. IFNγ-treated macrophages were infected with *B. cenocepacia*(pDsRedT3) and bacterial intracellular localization was followed over time. The quantification of the colocalization between the BcCV and intracellular markers was performed by calculating the PCC as indicated in Materials and methods. As early as 3* *h post-infection, 27% of the late endosome marker Rab7 (PCC: 0.268), 22% of the late endosome/lysosome LAMP1 (PCC: 0.2158), and the 35.5% of the class II MHC molecules (PCC: 0.3551) colocalized partially with the BcCVs (Figure 3). Therefore, the BcCVs can acquire class II molecules, suggesting they could have similar properties as MIIC where bacterial antigens are processed, which also include being a non-acidic intracellular compartment (Figure 4(A)). A biochemical experiment examining BcCVs purified by sucrose density gradient also showed that the majority of live *B. cenocepacia* is localized in fractions with low lysosomal activity (as defined by measuring the levels of β-galactosidase activity) and reduced LAMP1 expression (Fractions 10-12, Figure 4(B)). These data support the notion that BcCVs share properties of an MIIC compartment whereby bacterially-derived peptides are loaded into class II molecules and stimulate specific CD4^+^ T-cell responses.

**Figure 5.**
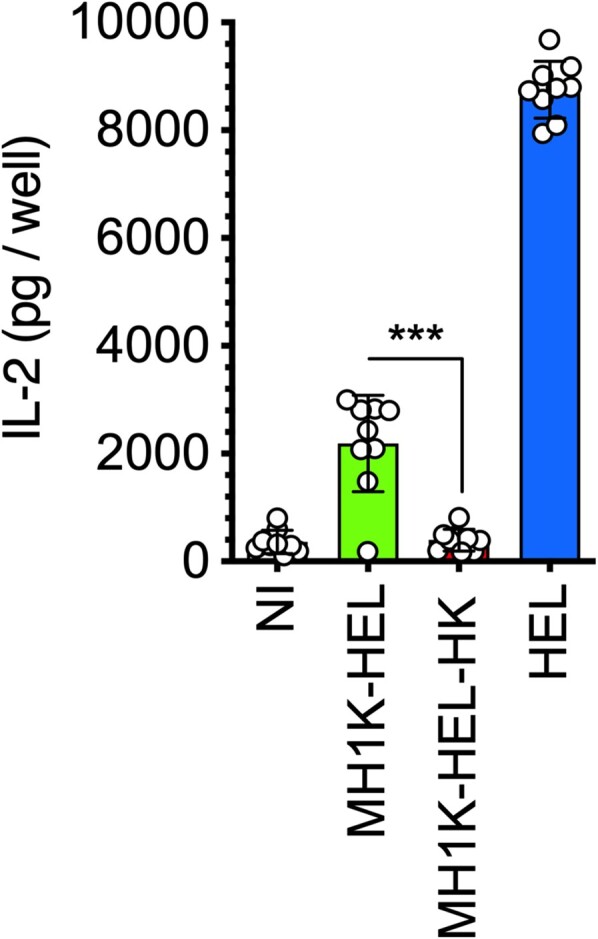
Macrophages infected with *B. cenocepacia* present bacterial antigens to CD4^+^ T-cells. Macrophages from C3H/HeJ mice were pre-treated with 300 U/ml of IFNγ prior to infection with *B. cenocepacia*-p*zmpA*-HEL (MH1K-HEL) at MOI of 50 during 1* *h. Infected macrophages were co-cultured with C-10 cells, a CD4^+^ T-cell hybridoma during 24* *h. Gray bar indicates the response of the C-10 hybridoma with non-infected cells (NI). Green bar indicates macrophages infected with MH1K-HEL, red bar indicates macrophages infected with heat-killed MH1K-HEL (MH1K-HEL-HK). Blue bar indicates the response of C-10 against macrophages pulsed with 250* *μg/ml of HEL. The graph represents the results of 3 independent experiments in triplicate (*n* = 9), plotted as mean ±SD and analyzed by the paired t-test. **, *p*=0.0038; ns, non-significant.

To further support this hypothesis, IFNγ-treated macrophages were infected with *B. cenocepacia* and assessed for their ability to present antigens to CD4^+^ T-cells. For specific-antigen recognition, we used a plasmid encoding the fusion protein ZmpA-HEL_48-61_, the HEL-peptide is presented exclusively by class II MHC molecules of haplotype I-A^k^, (hereafter, HEL) [41] to CD4^+^ T-cells. ZmpA is a secreted *Burkholderia* metalloprotease, presumably released into the BcCV by the Type II Secretion System (T2SS) [26,42]. If BcCVs share MIIC properties, we would expect that ZmpA could be processed by luminal resident proteases. This idea was tested by using IFN-γ-treated BMDM from C3H/HeJ (Haplotype I-A^k^) mice that were infected with *B. cenocepacia*-HEL and co-cultured with C-10, a CD4^+^ T-cell hybridoma, which recognizes the HEL_48-61_ peptide loaded by I-A^k^ class II molecules [31]. IL-2 released by C-10 hybridoma was quantified in the co-culture supernatants. The results indicate that viable macrophages infected with live *B. cenocepacia*-HEL can present bacterial antigens to CD4^+^ T-cells in the context of class II MHC molecules (Figure 5). Indeed, macrophages infected with heat-killed *B. cenocepacia*-HEL (MH1K-HEL-HK) did not present the ZmpA-HEL antigen to CD4^+^ T-cells. Therefore, only macrophages containing viable bacteria can present the antigen since only viable bacteria produce and translocate the fusion protein outside the BcCV into the cytosol by the combined actions of the T2SS and T6SS, as previously described [26] where it may be processed and presented to CD8^+^ T-cells. Together, the experiments presented here indicate that BcCVs acquire the ability to process and present bacterial antigens to T-cells.

### Macrophages infected with B. cenocepacia present bacterial antigens to CD8^+^ T cells

**Figure 6.**
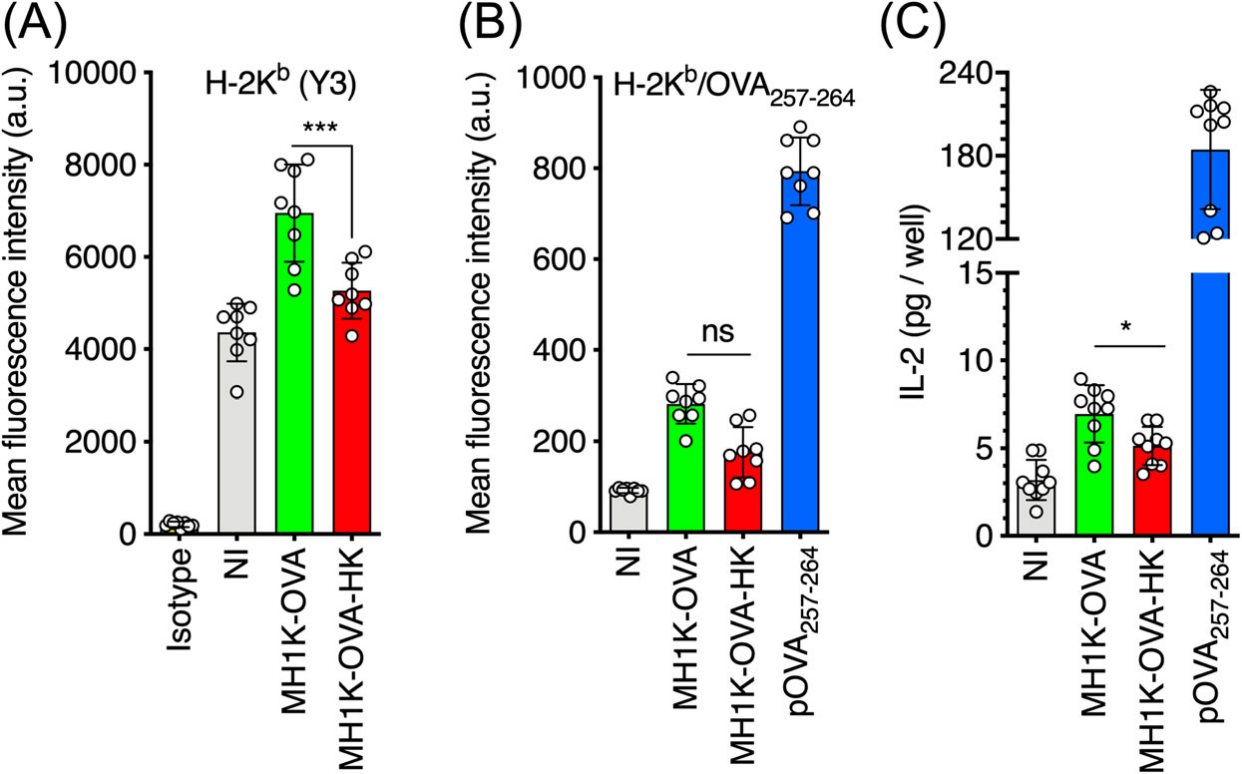
Macrophages infected with *B. cenocepacia* present bacterial antigens to CD8^+^ T-cells. Macrophages from C57Bl/6 mice were pre-treated with 300 U/ml of IFN*γ* and infected with *B. cenocepacia*-p*zmpA*-OVA (MH1K-OVA) or heat-killed (MH1K-OVA-HK) at MOI of 50 during 1 h. (A) Quantification of class I MHC H-2K^b^/peptide complexes at 24 h post-infection. (B) Quantification of class I MHC molecules H-2K^b^/pOVA_257-264_ complexes at 24 h post-infection. (C) Infected macrophages were co-cultured with the RF33.70, a CD8^+^ T-cell hybridoma for 24 h. White bar indicates isotype antibody (A). Gray bars indicates non-infected cells (NI) (A, B), and response of RF33.70 hybridoma with non-infected cells (C). Green bars indicate macrophages infected with MH1K-OVA, red bars indicates macrophages infected with MH1K-OVA heat-killed (MH1K-OVA-HK). Blue bar indicates macrophages pulsed with100 ng/ml pOVA_257-264_ (B) and the response of RF33.70 hybridoma against macrophages pulsed with 100 ng/ml of pOVA_257-264_ (C). The results presented in panel A and B were obtained from 4 independent experiments, each one in duplicate (*n* = 8), and the result presented in panel C was obtained from 3 independent experiments, each one in triplicated (*n* = 9) and analyzed by one-way ANOVA with the Turkey multiple comparison test. The bars were plotted as mean ± SD. *, *p*<0.05; ns, non-significant.

To investigate whether *B. cenocepacia*-infected macrophages can also present specific bacterial antigens by class I MHC molecules to CD8^+^ T-cells, we constructed a plasmid encoding the fusion protein ZmpA-OVA_257-264_ (hereafter OVA). The OVA-peptide is specifically presented by MHC class I, haplotype H-2K^b^ [43]. The T6SS of *B. cenocepacia* affects the integrity of the BcCV membrane allowing ZmpA to gain access into the cytosol [26]. Cytosolic ZmpA could be processed by the proteasome and follow the endogenous pathway of antigen processing and presentation. To test this hypothesis, IFN*γ*-treated BMDM from C57Bl/6 (haplotype H-2K^b^) were infected with *B. cenocepacia*-OVA, and the level of class I MHC molecules (Figure 6(A)) and the H-2K^b^/pOVA_257-264_ complex arising from *B. cenocepacia* processing (Figure 6(B)) were quantified by flow cytometry. Expression of MHC class I molecules increased in macrophages infected with *B. cenocepacia* (Figure 6(A)). In contrast, specific H-2K^b^/pOVA_257-264_ complexes did not increase significatively at the cell surface of macrophages infected with *B. cenocepacia*-OVA (Figure 6(B)). We explored the ability of *B. cenocepacia*-OVA-infected macrophages to present specific-bacterial antigens to T-cells by co-culturing infected macrophages with RF33.70, a CD8^+^ T-cell hybridoma that specifically recognizes the OVA_257-264_ peptide [33] loaded by H-2K^b^ molecules. IL-2 released by RF33-70 hybridoma in the supernatant indicates that *B. cenocepacia*-OVA-infected macrophages can present bacterial antigens by class I MHC molecules to CD8^+^ T-cells (Figure 6(C)). Together, these results suggest that *B. cenocepacia*-infected macrophages can also process and present bacterial antigens by class I MHC molecules to CD8^+^ T-cells.

### B. cenocepacia-infected macrophages release processed bacterial peptides into the extracellular medium that stabilize empty class I MHC molecules

**Figure 7.**
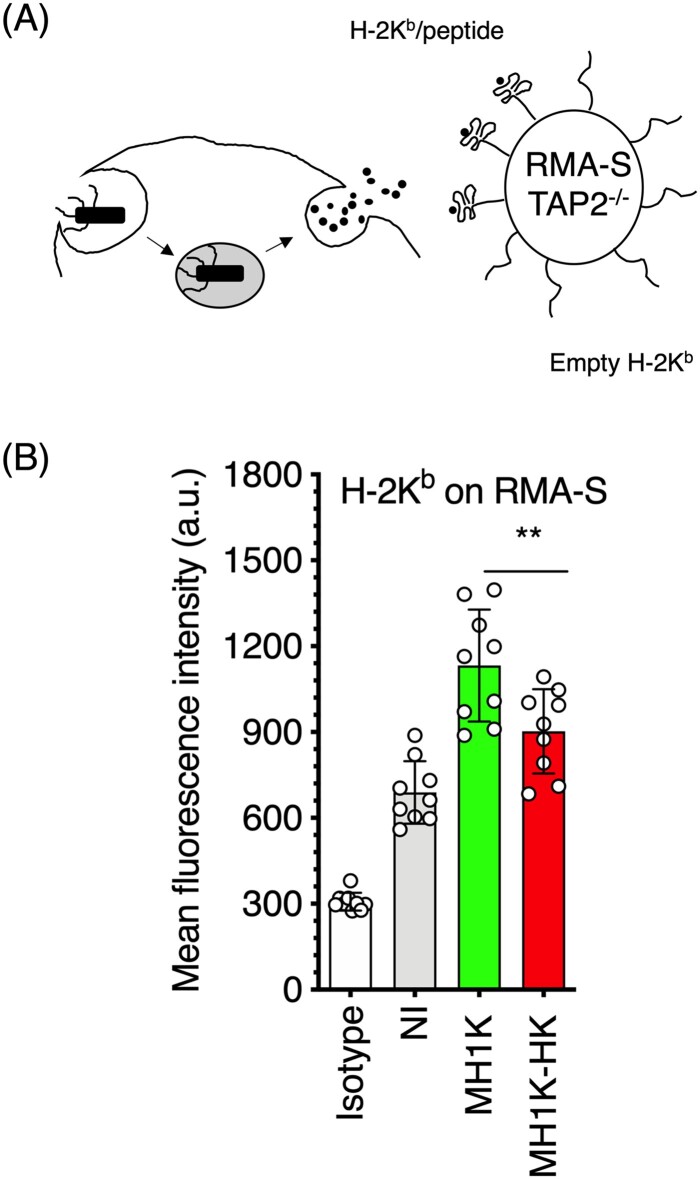
Macrophages infected with *B. cenocepacia* release products of bacterial processing into the extracellular media. Macrophages from C3H/HeJ mice (H-2K^k^) were pre-treated with 300 U/ml of IFNγ and infected with *B. cenocepacia* (MH1* *K) at a MOI of 50 during 1* *h. (A) Infected macrophages were co-cultured with RMA-S (H-2K^b^) cells at a 1:2 ratio during 24* *h. (B) Class I MHC H-2K^b^/peptide complexes were quantified by flow cytometry with Y3 (anti-H-2K^b^/peptide) monoclonal antibody. White bar indicates isotype antibody (B). Gray bar indicates the quantification of class I MHC/peptides on RMA-S cells cocultured with non-infected macrophages (NI), green bars, RMA-S cells co-cultured with macrophages infected with live (MH1* *K) and red bars, RMA-S cells co-cultured with macrophages infected with heat killed (MH1K-HK) bacteria. The results were obtained from 3 independent experiments, each one in triplicate (*n* = 9), plotted as the mean of fluorescence intensity ± SD, and analyzed by one-way ANOVA with the Turkey multiple comparison test. *, *p*=0.0187.

When infected with *B. cenocepacia*, IFNγ-treated macrophages show reduced intracellular bacterial load (Figure 2(A)). Therefore, we also investigated if *B. cenocepacia*-infected macrophages could release peptides from processed bacterial antigens into the extracellular medium. For these experiments, *B. cenocepacia*-infected macrophages from C3H/HeJ mice were co-cultured with the TAP-2^-/-^ RMA-S cells that express empty class I MHC molecules on their plasma membrane (Figure 7(A)) [44]. After co-culture of RMA-S cells with infected macrophages for 24* *h, RMA-S cells were analysed by flow cytometry to determine the amount of MHC class I molecules with stable conformation [23]. The results indicated a higher level of class I MHC/peptide complexes on RMA-S cells’ surface when co-cultured with infected macrophages (Figure 7(B)), suggesting that IFNγ-activated macrophages infected with viable bacteria can release bacterial peptides (derived from *B. cenocepacia* processing) that stabilize empty class I MHC molecules of bystander cells. In contrast, heat-killed *B. cenocepacia-*infected macrophages did not efficiently release bacterial peptides derived from antigen processing.

## Discussion

**Figure 8.**
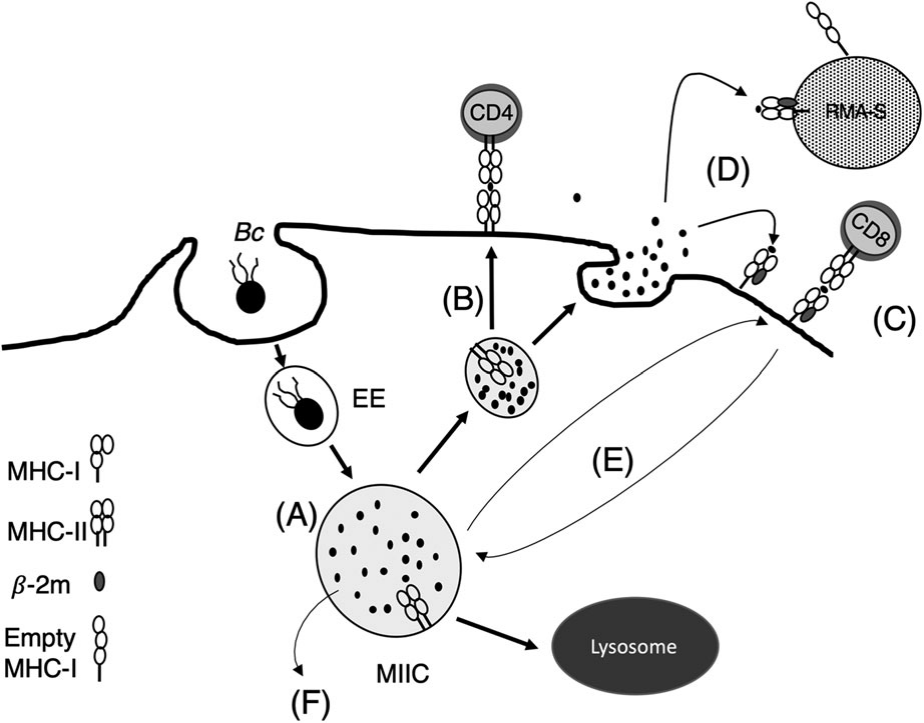
Model of antigen processing and presentation of *B. cenocepacia* by class I and class II MHC molecules to T-cells. (A) MIIC in which live *B. cenocepacia* is processed. (B) Bacterial antigens are recognized by CD4^+^ T-cells or (C) CD8^+^ T-cells. (D) Products of bacterial processing are released to the extracellular media. (E) Recycling of class I MHC from plasma membrane to the MIIC. (F) Translocation of bacterial antigens from the MIIC to the cytosol.

In this study, we demonstrate that IFNγ-activated macrophages infected with *B. cenocepacia* can process and present bacterial antigens to T-cells by both class I and class II MHC molecules. Intracellular *B. cenocepacia* survive in a vacuole (BcCV) that delays fusion with lysosomes, preventing luminal acidification for at least 6* *h post-infection [8] and also exhibiting proteolytic activity [26]. The MIIC compartment that processed antigens also contains low lysosomal activity and its membrane is decorated with class II MHC molecules, Rab7 and LAMP1 [39,40]. Our results indicate that the BcCV, containing live *B. cenocepacia*, acquires Rab7, LAMP1 and class II MHC molecules before lysosomal fusion. The BcCV is a not acidic compartment that also has low lysosomal activity, both features suggesting that the BcCV resembles the main features of MIIC before lysosomal fusion. Therefore, it is conceivable that bacterially-derived peptides within the BcCV are loaded into class II MHC molecules until eventually the fusion with lysosomes leads to complete antigen degradation (Figure 8(A))*.* This model is consistent with our observation that compared to untreated cells, fewer bacteria (21 ± 17%) survive in IFNγ-treated macrophages. IFNγ pre-activated macrophages have an increased ability to process exogenous antigens due to increased expression of several IFNγ-induced endosomal proteases [36]. Likewise, early endosomes are also involved in exogenous antigen processing [45] due to the presence of IFNγ-induced lysosomal thiol reductase (GILT) [46]. In addition, IFNγ also promotes autophagy, a process by which macrophages control the proliferation of intracellular *B. cenocepacia* [47]. Indeed, autophagy increases the efficiency of antigen presentation by MHC class II molecules [48]. It has been reported that CD4* *T-cells that recognize flagellin from *B. pseudomallei* can also recognize IFNγ-treated macrophages infected with viable *B. cenocepacia* J2315. Further, IFNγ-treated macrophages infected with heat-killed J2315 induce a stronger response than macrophages infected with live-J2315. This is expected since flagellin is an antigen present both in live and dead bacteria [49]. In contrast, our results show that IFNγ-treated macrophages infected with live *B. cenocepacia* present bacterial antigens more effectively than macrophages infected with dead bacteria, in agreement with the expression of ZmpA-pHEL_48-61_ in live *B. cenocepacia*.

APCs can also process and present antigens by class I MHC molecules using alternative pathways. One of them, defined as cross-presentation [50,51], involves the transport of engulfed exogenous antigens from the vacuolar lumen into the cytosol (Figure 8(F)) and subsequently follows the classical pathway of antigen processing and presentation [21]. Another pathway involves the processing of the engulfed exogenous antigen within a vacuole [52]. We posit that the BcCV could be a vacuole for antigen processing. This is supported by our results demonstrating that macrophages infected with *B. cenocepacia* can present bacterial antigens by class I MHC molecules to CD8^+^ T-cells. The peptides generated by this mechanism could also stabilize post-Golgi class I MHC molecules (Figure 8(E)). As shown for other vacuoles, this pathway likely requires a peptide dissociation/exchange directly into the vacuole of antigen processing [53]. Further, peptides generated from the exogenous antigen processing could be released into the extracellular medium in which empty class I MHC molecules of self-cell or bystander-cells can be loaded by these peptides [23,34]. Our results indicate that *B. cenocepacia-*infected macrophages process the bacterium and the generated peptides are released into the extracellular media, stabilizing empty class I molecules of bystander-cells (Figure 8(D)).

In summary, this work illustrates the ability of *B. cenocepacia*-infected macrophages, when activated with IFNγ to process and present *B. cenocepacia* antigens by class I and class II MHC molecules to CD8^+^ and CD4^+^ T-cells, respectively. We also demonstrate that the BcCV plays a key role in this process by acquiring properties similar to the MIIC. The role of specific CD4^+^ and CD8^+^ T-cells during the induction of protective immunity against *B. cenocepacia* remains to be explored.

## Supplementary Material

Rosales-Reyes_TEMI-2020-0804-Supplementary_Table_1_final.doc
