# Supplementary material for: Interferon-gamma-activated macrophages infected with Burkholderia cenocepacia process and present bacterial antigens to T-cells by class I and II major histocompatibility complex molecules
Source: Emerg Microbes Infect. 2020 Sep 17;9(1):2000–12. doi: 10.1080/22221751.2020.1818632 (PMC7534305; doi:10.1080/22221751.2020.1818632)
Supplement: Rosales-Reyes_TEMI-2020-0804-Supplementary_Table_1_final.doc [file TEMI_A_1818632_SM7187.doc]

**Supplementary Table 1**. Bacterial strains and plasmids used in this study


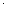

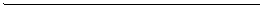


| Strain or Plasmid | Relevant Characteristics |
| --- | --- |
|  |  |
| *Burkholderia cenocepacia* | K56-2, D*amrABC* (BCAL1674- |
| MH1K | 1676); Gm^S^ |
| *Escherichia coli* DH5a | F- f80*lacZ* M15 *endA1 recA1* |
|  | *supE44 hsdR17*(r_K_-m_K_+) *deoRthi*-1 |
|  | *nupG supE44 gyrA96 relA1* |
|  | D(*lacZYA*-argF) U169, l^–^ |


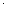


Source or

Reference


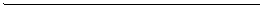


[24]

Laboratory stock

| pdsRedT3 | Expresses Red fluorescent protein, | [26] |
| --- | --- | --- |
|  | Cm^R^ |  |
| pSCrhaB2 | Cloning vector inducible with | [54] |
|  | rhamnose, *ori*pBBR1, *rhaR rhaS* |  |
|  | *PrhaB* Tp^R^ mob+ |  |

pSCR-ha-*zmpA*-HEL48-61 (pDA195)

pSC-ha-*zmpA*-OVA254-267 (pDA196)

pRK2013

| Expresses the protein ZmpA fused | This study |
| --- | --- |
| C-terminally to HEL48-61 peptide; |  |
| Tp^R^ |  |
| Expresses the protein ZmpA fused | This study |
| C-terminally to OVA254-267 peptide; |  |
| Tp^R^ | [55] |
| *oricolE1*, RK2 derivative, Kan^R^, |
| mob^+^, tra^+^ |  |


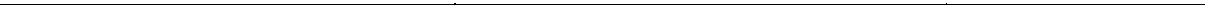


Amp, ampicillin; Cm, chloramphenicol; Gm, gentamicin; Kan, kanamycin; Tp, trimethoprim;

Tet, tetracycline.
